# Sense of Ethnic Belonging: Relation With Well-Being and Psychological Distress in Inhabitants of the Mapuche Conflict Area, Chile

**DOI:** 10.3389/fpsyg.2020.617465

**Published:** 2021-01-11

**Authors:** Felipe E. García, Loreto Villagrán, María Constanza Ahumada, Nadia Inzunza, Katherine Schuffeneger, Sandra Garabito

**Affiliations:** ^1^Facultad de Ciencias Sociales y Comunicaciones, Universidad Santo Tomás, Concepción, Chile; ^2^Departamento de Psiquiatría y Salud Mental, Facultad de Medicina, Universidad de Concepción, Concepción, Chile; ^3^Departamento de Psicología, Facultad de Ciencias Sociales, Universidad de Concepción, Concepción, Chile

**Keywords:** identity, ethnic belonging, discrimination, well-being, mental health, Mapuche

## Abstract

Research has shown that experiences of discrimination cause harm to the health and well-being of people. In terms of the identity of members of a group, a positive evaluation of that group might involve devaluing the out-group as a way of raising the endo-group, causing discrimination toward the out-group. In the Chilean context, the Mapuche people have historically suffered discrimination and violations of their rights. The aim of this study was to assess the relationship between Collective Identity, perceived experiences of discrimination, psychological well-being and distress in the inhabitants of the Mapuche conflict zone according to their sense of belonging to their ethnic group (Mapuche, Mestizo, Caucasian). This descriptive, correlative, and cross-sectional study involved 200 participants, including 94 men (47%), and 106 women (53%), between the ages of 18 and 83 years old (*M* = 39.02; *SD* = 13.45), who had lived for at least 1 year in communities in the Araucanía Region. The sample was stratified according their sense of ethnic identity, including 30% Mapuche, 33.5% Caucasian, and 36.5% Mestizo. The results show that participants with a sense of Mapuche ethnicity experienced more instances of discrimination, had a greater sense of collective identity, and that they also supported the Mapuche social movement and its methods. Based on evidence that well-being is directly related to collective identity, the study undertook a regression analysis of emotional distress and the psychological well-being of participants. The interaction between experiences of discrimination and collective identity has a significant influence. Collective identity and experiences of discrimination in themselves as well as the interaction between them, predict psychological well-being. The results suggest that the importance of the Mapuche group’s identity phenomena are related to a broad socio-historical context that leads them to identify themselves as a collective in conditions of inequality. This relationship between well-being and collective identity could be explained by their sense of cultural belonging, which can be a factor in protecting mental health.

## Introduction

Chile has three majority ethnic groups. Among them, the Mapuche live mainly in rural areas but have started to integrate into city communities. There are also Caucasians, whose physical features contrast sharply with the Mapuche, for example, due to their lighter complexion. The third main majority group is Mestizos, who have both Mapuche and European heritage (Corporación Latinbarómetro, 2011).

According to the last census (Instituto Nacional de Estadísticas, 2017), 12.8% of the Chilean population is considered to belong to indigenous people. Of that percentage, 79.8% consider themselves Mapuche. Despite this large number, studies show that when Chilean Caucasian or Mestizo populations have direct contact with Mapuche people they experience significant, though not necessarily explicit, levels of prejudice, and discrimination (Merino et al., 2009; Ramírez et al., 2016), as perceived by the Mapuche population (Instituto Nacional de la Juventud, 2012). Due to this discrimination, the Mapuche population experience psychological damage, feelings of anger, shame, and powerlessness, along with actions that involve self-protection, self-control, or confrontation (Merino et al., 2009, 2020). Furthermore, data show that the suicide rate in the Mapuche population is higher than the non-Mapuche population, with increased instances between 2004 and 2006 (Centro Latinoamericano y Caribeño de Demografía, 2011), and 2006 and 2010 (Guajardo, 2017).

Discrimination against the Mapuche people is part of a historical process that dates back more than a century, involving a violation of rights that continues today. Throughout this process, the Mapuche people have been dispossessed of a large amount of their land and are repressed by the Chilean state. The so-called “Mapuche conflict” corresponds with the ethnic and territorial struggle that involves a complex confrontation between these people, the state, the forestry industry, and agricultural landowners (Meza, 2015).

Various reports document the situation of rights violations among the Mapuche (Stavenhagen, 2003; Instituto Nacional de Derechos Humanos Chile, 2013). There has been structural violence, they have been excluded from education and labor, and lack access to basic services, all of which means there is poor nutritional health and lower incomes among the Mapuche (Rojas and Lobos, 2016). This exclusion is accentuated by perceived discrimination (Tricot, 2008; López, 2016).

The Chilean government has recognized the inequality in welfare and development experienced by indigenous peoples (Ministerio de Desarrollo Social Chile, 2020), considering them a minority priority group in social policies. In 1993 the National Corporation for Indigenous Development (CONADI) was created, whose mission is to promote, coordinate, and execute the actions of the State in promoting the integral development of indigenous individuals and communities, especially in economic, social, and cultural spheres, and to encourage their participation in national life (Corporación Nacional de Desarrollo Indígena, 2020). However, these actions have not diminished the intensity of the conflict which has resurfaced in recent years as a result of police repression, including the assassination of a Mapuche community member by the police, which triggered a wave of protests in 2018 in different regions of the country (Calfio et al., 2019). In 2020, a “Chilean” population group confronted and forced a group of Mapuche civil rights protesters to vacate public buildings that were occupied as part of a protest (Rojas Pedemonte and Bresciani, 2020).

It has been found that the discrimination experienced by some groups causes harm to health and well-being. Some meta-analyses have found that perception of discrimination impacts physical and mental health, producing high levels of stress, unhealthy behaviors, and psychopathological symptoms (Pascoe and Smart, 2009; Bardol et al., 2020). This adverse effect is accentuated when discrimination is directed toward the stable attributes of a group, for example, their ethnic or national origin, gender, religion, or place of residence (Soberanes, 2010; García et al., 2017; Hynie, 2018).

People who feel discriminated against because of their ethnicity may exhibit negative emotional states, such as stress, aggression, and depressive symptoms (García et al., 2017). Experiences of discrimination are manifested in behaviours such as mistreatment, suspicions about their morals or skills, and their presence may even be ignored (Segato, 2011). This behavior affects basic aspects of the everyday life of the person/group who is discriminated against, impacting their interpersonal relations, employability, and access to housing and daily life (López et al., 2008; Ruiz, 2015).

Discrimination is characterized by behaviors of action or omission that deny equal treatment of members of the out-group, which are explained through processes related to social identities, like categorization processes, stereotypes, and prejudice (Tajfel and Forgas, 1981; Dovidio and Gaertner, 1986). This is because group identity can moderate the relationship between perceived discrimination and health. The factors linked to group identity processes and inter-group power relations have been proposed as key mechanisms in the reinforcement and maintenance of discrimination (Dovidio et al., 2010).

Belonging to a group involves the positive or negative assessment of shared characteristics, which form a person’s social identity (Tajfel and Forgas, 1981). Social identity is defined as that part of the self-concept derived from the knowledge of belonging to a social group together with the emotional and evaluative meaning associated with that belonging (Tajfel and Turner, 1986). In collective contexts, identity becomes very relevant, as the individual evaluates themself and other people in terms of their group membership (Javaloy, 1993).

The positive aspects of group identity have been associated with subjective well-being (Smith and Silva, 2011; Ye and Ng, 2019). Ethnic identity can provide a coping strategy in the face of discrimination and a protective factor for mental health (Mossakowski, 2003). Groups that maintain reciprocal support systems provide a peer-support network for members in times of crisis such as social or natural disasters. Conversely, when people only deploy individual coping mechanisms, the support received will be less or non-existent (Cicognani et al., 2008; Gallagher et al., 2019).

Studies in contexts other than Latin America have found links between high levels of ethnic identity and low symptoms of depression, thoughts of suicide, and history of suicide attempts (Cheng et al., 2010). In the national context, one study found that there is a positive relationship between Mapuche ethnic identity, well-being, and resilience (Pilquimán et al., 2020). The results of these studies reinforce the idea that positive ethnic identity is a protective factor in the emergence of depressive symptoms and suicidal ideation (Tereucán et al., 2017). It has also been suggested that a person’s link to ethnicity could also provide greater resources that enable them to address the negative effects of acculturative stress and discrimination (Cheng et al., 2010).

One aspect of social identity is collective identity (Simon and Klandermans, 2001), which has the following components (Melucci, 2004): (a) there are cognitive definitions of the group’s particularities, meanings, and fields of action (for example shared language, rituals, and cultural practices); (b) it forms a network of relationships between its actors, through common negotiation and decision-making processes, through shared communication channels; and, (c) that there is an emotional investment among members of the endo-group that facilitates belonging to the group, i.e., the emotional expenditure.

On the other hand, a politicized collective identity implies awareness and commitment among group members to participate in power struggles (Simon and Klandermans, 2001; Klandermans, 2013). These struggles aim at the gradual transformation of the group’s relationship with its social environment. According to a meta-analysis by Van Zomeren et al. (2008) a politicized collective identity is the most important predictor of collective action and has larger effect dimensions. The variables of injustice, identity, and effectiveness predict collective action in a similar way, but with a moderate effect size.

Perceptions of injustice and collective distress encourage participation in social movements or collective opposition actions against a dominant group (Fominaya, 2010; Klandermans, 2013). This includes support for violent political action (Páez et al., 2013; Wohl et al., 2014), providing participants with a sense of belonging and group identity (Javaloy, 1993).

Given the relationship between experiences of discrimination, collective identity, distress, and emotional well-being, as well as the protective role that collective identity appears to have in mental health, this paper aimed to evaluate these variables in inhabitants of the Mapuche conflict zone according to their sense of belonging to their ethnic group (Mapuche, Mestizo, Caucasian) and the relationship between them. The study hypothesized that: (H1) experiences of discrimination have a positive relationship with distress and a negative relationship with well-being; (H2) that experiences of discrimination, collective identity, distress, and well-being predict participation in social movements; and, (H3) that collective identity has a buffering effect on the relationship between experiences of discrimination with distress and psychological well-being.

## Methodology

### Design

The present study used a descriptive and correlational research design, the data were collected in a single time frame, corresponding to a cross-sectional study.

### Participants

The study involved 200 participants, including 94 men (47%) and 106 women (53%), aged between 18 and 83 years old (*M* = 39.02; *SD* = 13.45), who had resided for at least 1 year in communes belonging to the Araucanía region. Of these, 156 (78%) belonged to urban areas and 44 (22%) to rural areas. Stratified sampling was established so that the three predominant ethnic/racial groups in the area were represented equitably; thus the participating group was composed of 60 Mapuche (30%), 67 Caucasians (33.5%), and 73 Mestizo (36.5%).

The power of the study was calculated considering the sample size, using the program G-power, considering a medium effect size, an alpha error of 0.05 and four predictors for the last regression, obtaining a power of 0.99.

### Instruments

#### Collective Identity

Collective identity was measured by the Collective Identity Scale (based on Van Zomeren et al., 2013; García et al., 2017), which consists of six items that measure group identification, e.g., when a person “feels that they have a lot in common with other people who belong to their ethnic or racial group [Mapuche, Caucasian (white), Mestizo or other]” and has a politicized sense of collective identity, e.g., a person who “identifies with other people who participate in movements supporting their ethnic or racial group [Mapuche, Caucasian (white) or Mestizo].” They responded on a Likert scale from one (“none”) to seven (“a lot”). High scores indicated high identification with the group and with the group’s demands. In the present study, the scale showed an internal consistency of α = 0.95.

#### Discrimination

The short scale of discrimination experiences was used (Landrine and Klonoff, 1997; Smith-Castro, 2010), which consists of six items reporting the frequency with which they have experienced different situations such as disrespect, jokes, unfair treatment by bosses or colleagues, lack of employment opportunities, and physical aggression linked to the ethnic group. An example is: “How often have you heard people making jokes (pranks) about people who belong to your ethnic or racial group [Mapuche, Caucasian (white), Mestizo or other]?” In this study, the scale showed an internal consistency of α = 0.78.

#### Psychological Well-Being

The Mental Health Continuum-Short Form (MHC-SF) scale, developed by Keyes (2005), was used to measure psychological well-being (Spanish version by Aragonés et al., 2011). This instrument consists of 14 items that measure different aspects of well-being (e.g., “during the last month, how often have you felt satisfied with life?”), which allows us to give a general measure of well-being. This scale is answered by a six-point Likert-type scale, from one (“never”) to six (“every day”). In this study, internal consistency was obtained from α = 0.84.

#### Distress

The Chilean version of Goldberg’s 12-item General Health Questionnaire [GHQ-12] Goldberg (1978) by Herrera and Rivera (2011) was used. This scale is designed to detect mental health problems (e.g., “Have you been feeling unhappy and depressed?”). It is answered on a 4-point Likert scale, ranging from zero to three. The present study showed satisfactory internal consistency (α = 0.80).

#### Socio-Demographic and Psycho-Social Questionnaire

A socio-demographic questionnaire was developed to collect information on age, sex, place of residence, marital status, and sense of belonging to an ethnic group (Mapuche, Mestizo, or Caucasian). Psycho-social questions were also included to find out the degree of support or rejection of the Mapuche social movement, the protest methods used, the state’s response to this movement, and the extent to which they have been affected by the Mapuche movement.

### Procedure

A pilot test was developed and applied to a total of six adults with primary education to evaluate the understanding of the items and the time of application. The pilot test was conducted in a range of 10–30 min and some participants expressed problems in understanding some instructions or items. As a result, the instructions on the Discrimination and Distress Experience Scale were modified to make them clearer, and the expression “you” (Spanish “tú,” informal) was changed to “you” (Spanish “usted,” formal) on the Collective Identity Scale. We also made contact with residents of the Araucanía Region, who completed questionnaires regardless of their degree of support for or rejection of the Mapuche social movement, as a way of balancing each ethnic group in terms of size. Therefore, the sampling was intentional by quotas. With this in mind, the surveys were applied individually. Before they participated, we explained the objectives of the study to each participant and informed them about confidentiality, making it clear that this was an anonymous and voluntary process. They were also required to sign an informed consent letter. Of the total number of people consulted, 22 refused to participate on the grounds of lack of time or mistrust. Finally, this study was approved by the Ethics Commission of Saint Thomas University, with resolution number 16–18, in the year 2018.

### Data Processing and Analysis

First, we conducted a descriptive analysis of criterion variables together with ANOVA tests to compare them between the ethnic groups. For *post hoc* contrasts, Tukey’s HSD contrast was used. Subsequently, we calculated Pearson correlations to evaluate the relationship of the interest variables and finally, we conducted several regression models and hierarchically presented them to evaluate possible differences among models. All the analyses were conducted with the SPSS v.21 (IBM Corp, 2011) statistical software and the PROCESS macro, following the criteria proposed by Hayes (2013). The power of the study was calculated considering the sample size, using the program G-power, considering medium effect size, an alpha error of 0.05, and four predictors for the last regression, obtaining a power of 0.99.

## Results

Table 1 shows that people from the Mapuche group experienced more instances of discrimination and that they had a strong collective identity, with higher support for the Mapuche social movement, including the methods used by this movement. Responses also indicated that they more often disagreed strongly with the state’s actions to confront the social movement compared to the Caucasian and Mestizo groups. The latter two do not differ in any of the variables. There were no differences between the groups in terms of emotional well-being and distress.

**TABLE 1:** Descriptive statistics of the study variables in the total group and each ethnic and racial group.

**Variable**	***Min***	***Max***	**Total *n* = 200**		**Mapuche [MA] *n* = 60**		**Caucasian [CA] *n* = 67**		**Mestizo [ME] *n* = 73**		***F* value**	**Comparisons**
			***M***	***SD***	***M***	***SD***	***M***	***SD***	***M***	***SD***		
Discrimination	6	26	8.92	3.55	11.98	3.90	7.64	2.66	7.56	2.18	44.247***	MA > CA and ME
Collective identity	6	36	20.48	10.34	25.39	10.07	18.95	10.67	17.85	8.95	10.303***	MA > CA and ME
Support to the Mapuche social movement	1	10	4.61	3.09	6.90	2.57	3.10	2.46	4.03	2.91	35.426***	MA > CA and ME
Support for the methods used by the Mapuche social movement	1	10	2.93	2.76	4.74	2.99	1.99	2.19	2.24	2.27	23.963***	MA > CA and ME
Support for the actions of the state	1	10	4.55	3.30	3.02	2.48	4.66	3.17	5.79	3.53	13.167***	CA and ME > MA
Emotional distress	0	11	2.26	2.33	2.19	2.35	2.53	2.42	2.07	2.24	0.671	MA = CA = ME
Psychological well-being	33	84	61.91	10.44	60.77	11.41	63.44	10.39	61.51	9.63	1.049	MA = CA = ME

Comparison of groups through ANOVA. ***p < 0.001.

Correlations were carried out to examine H1 and H2. Table 2 shows the correlation between the variables. Concerning the first hypothesis, that experiences of discrimination have a positive relationship with distress and a negative relationship with well-being, results show that emotional distress was positively and well-being negatively related with discrimination, as expected, but that correlations were not significant. The second hypothesis posits that experiences of discrimination, collective identity, distress, and well-being predict participation in social movements, and correlation results confirm this. Discrimination is associated with a collective identity, and both variables are positively correlated, with support for the Mapuche movement and support for the methods used by the Mapuche movement. They are negatively related to support for the actions of the state in confronting the Mapuche movement.

**TABLE 2:** Pearson’s *r* correlations between study variables.

**Variable**	**2**	**3**	**4**	**5**	**6**	**7**
1. Discrimination	0.33***	0.30***	0.36***	−0.31***	0.13	−0.12
2. Collective identity	–	0.36***	0.36***	−0.20**	0.08	0.17*
3. Support of the Mapuche social movement		–	0.65***	−0.46***	0.00	−0.04
4. Support for the methods used by the Mapuche social movement			–	−0.46***	−0.03	−0.11
5. Support for the actions of the state				–	−0.11	0.06
6. Emotional distress					–	−0.17*
7. Psychological well-being						–

* *p* < 0.05; ** *p* < 0.01; *** *p* < 0.001.

To test the third hypothesis of moderation or that collective identity has a buffering effect on the relationship between experiences of discrimination with distress and well-being, a multiple linear regression analysis was carried out for the prediction of emotional distress. The first step considered experiences of discrimination and collective identity as predictors. The second step included the interaction between experiences of discrimination and collective identity. The analysis shows that moderation is significant. Moreover, including the interaction indicated that both interaction and collective identity have a significant influence, supporting H3 (see Table 3).

**TABLE 3:** Multiple linear regression on “well-being” with final model including interaction.

		**Non-standardized coefficients**		**Standardized coefficients**	***t* value**	***p* value**
		***B***	***SE***	**β**		
**Step 1**	***R*^2^ = 0.02; *p* = 0.161**					
	(Constant)	62.36	2.21		28.274	<0.001
	Discrimination	−0.58	0.22	−0.20	−2.644	0.009
	Identity	0.24	0.08	0.23	3.093	0.002
**Step 2**	***R*^2^ = 0.04; Δ *R*^2^ = 0.023; *p* = 0.038**					
	(Constant)	53.43	4.22		12.668	<0.001
	Discrimination	0.49	0.49	0.17	1.014	0.312
	Collective identity	0.66	0.19	0.65	3.514	0.001
	Discrimination X Identity	−0.05	0.02	−0.67	−2.470	0.014

**FIGURE 1:**
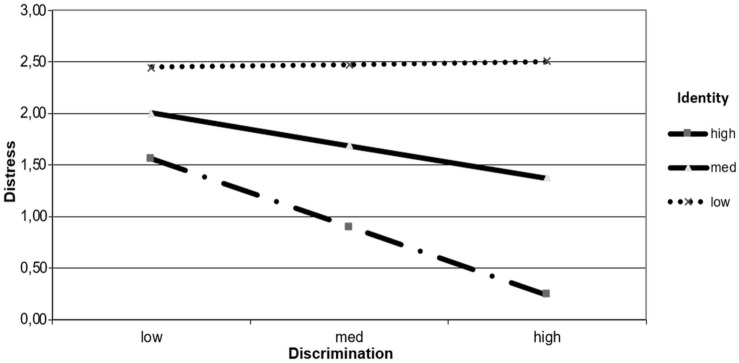
Distress explained by the interaction between experiences of discrimination and collective identity.

Indicating the results connected to the third hypothesis of moderation on distress, Figure 1 shows how low collective identity was associated with higher emotional distress regardless of experiences of discrimination. However, when collective identity was high and experiences of discrimination were also high, distress was reduced.

Table 4 presents a multiple regression that examines the third hypothesis, which is related to well-being. Multiple regression was carried out to predict psychological well-being, using the same predictors in step 1 and step 2, undertaken in the previous regression. In this case, both models are significant, but by including the interaction, the model improves its predictive capacity. In this case, both collective identity and experiences of discrimination and the interaction between the two predict psychological well-being.

**TABLE 4:** Multiple linear regression on “distress” with final model including interaction.

		**Non-standardized coefficients**		**Standardized coefficients**	***t* value**	***p* value**
		***B***	***SE***	**β**		
**Step 1**	***R*^2^ = 0.02; *p* = 0.161**					
	(Constant)	2.23	0.17		13.306	<0.001
	Discrimination	0.08	0.05	0.13	1.614	0.108
	Collective identity	0.01	0.02	0.03	0.444	0.658
**Step 2**	***R*^2^ = 0.04; Δ *R*^2^ = 0.02; *p* = 0.038**					
	(Constant)	2.35	0.17		13.416	<0.001
	Discrimination	0.10	0.05	0.15	1.962	0.051
	Collective identity	0.01	0.02	0.03	0.369	0.713
	Discrimination X Identity	−0.01	0.00	−0.15	−2.089	0.038

**FIGURE 2:**
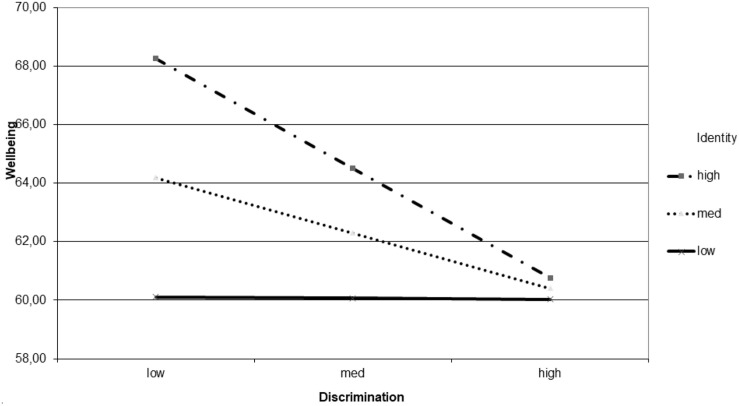
Well-being explained by the interaction between experiences of discrimination and collective identity.

Results relating to psychological well-being can be seen in Figure 2, which shows that when the collective identity was low, the level of well-being was also low. Similarly, when experiences of discrimination are high, the level of well-being was also low. However, if collective identity is high and experiences of discrimination are low, then well-being is high. In this case, the H3 of the buffering role of collective identity was not supported.

Finally, H2 was examined through a multivariate analysis using a multiple regression predicted variable of support for the Mapuche social movement. The predictors were experiences of discrimination, collective identity in the first step, with emotional distress and psychological well-being shown to be predictors in the second step. The model was significant, *F*(4,182) = 10,491; *p* < 0.001, with an *R*^2^ = 0.19 (*R^2^ adj* = 0.17). Table 5 shows that the significant predictors of support for the Mapuche movement are two collective variables: experiences of discrimination and collective identity; whereas the variables assessing individual responses, emotional distress, and psychological well-being, have no influence.

**TABLE 5:** Multiple linear regression on “support for the Mapuche movement.”

		**Non-standardized coefficients**		**Standardized coefficients**	***t* value**	***p* value**
		***B***	***SE***	**β**		
**Step 1**	***R*^2^ = 0.18; *p* = < 0.001**					
	(Constant)	4.59	0.20		22.725	<0.001
	Discrimination	0.21	0.06	0.24	3.331	<0.001
	Collective identity	0.08	0.02	0.28	4.013	<0.001
**Step 2**	***R*^2^ = 0.19; Δ *R*^2^ = 0.01; *p* = 0.500**					
	(Constant)	5.98	1.33		4.515	<0.001
	Discrimination	0.20	0.06	0.23	3.115	0.002
	Collective identity	0.09	0.02	0.30	4.143	<0.001
	Psychological well-being	−0.02	0.02	−0.07	−0.977	0.330
	Emotional distress	−0.07	0.09	−0.05	−0.805	0.422

## Discussion

This study focused on a sense of ethnic belonging among the inhabitants of the Araucanía region, an area where the “Mapuche conflict” has been most intense throughout history. It was considered relevant that the sample was composed of participants who identified with the three majority ethnic groups since the “Mapuche conflict” has also been proposed as a social process of “inter-ethnic conflict” (Cepal-Alianza Territorial Mapuche, 2012). This is evident in violent confrontations between “Chilean” and Mapuche civilians in 2020, which were interpreted by public opinion as racist actions (Rojas Pedemonte and Bresciani, 2020).

The results of an ANOVA test, which considered a sense of belonging to an ethnic group (Mapuche, Mestizo, Caucasian) as a factor, show that those who identify as belonging to the Mapuche group, report more experiences of discrimination and less support for the actions taken by the state in the face of Mapuche mobilizations. This information is consistent with previous studies which show that the Mapuche people continue to experience discrimination and exclusion in Chilean society (Merino et al., 2009, 2020; Ramírez et al., 2016).

One explanation for why people belonging to the Mapuche group feel more discriminated against than the other groups is related to the actions of the Chilean state. Currently, the Chilean state is concerned with promoting the development and participation of indigenous peoples (Corporación Nacional de Desarrollo Indígena, 2020), public policies consider the Declaration on the Rights of Indigenous Peoples (United Nations Development Programme, 2007), and there are social policies that consider indigenous peoples as a priority, among other inclusive actions. However, these actions have coexisted with state practices such as police repression (Aylwin, 2002), violation of rights, criminal accusations, the use of anti-terrorism laws to try Mapuche people accused of violent actions (Rojas and Gálvez, 2018), and the criminalization of protests in the media (Cepal-Alianza Territorial Mapuche, 2012; Droguett and Ojeda, 2015). The criminalization of protest is a phenomenon that has been studied in different contexts such as protests against the Argentinian government (Svampa and Pandolfi, 2004), the struggle for natural resources in Colombia (Olarte, 2014), migrants in Europe (Kubal, 2014), and political conflicts in Catalonia (Bernat and Whyte, 2020), among others. This phenomenon is linked to the actions of authoritarian governments, which exercise power through coercion, limiting political plurality, and restricting the political participation of the population (Linz, 1978). Among its effects is the assignment of a social stigma that labels the ex-group as “subversive” or a “public enemy” (Virseda et al., 2018).

The appearance and maintenance of a negative image of the Mapuche group can be understood through the cognitive construction of the image of the enemy (Martín-Baró, 1993). In this way, ideas, beliefs, and values that justify violence toward the group labeled as an “enemy” are promoted. When this comes from the state, processes of institutionalized lying and dehumanization seemingly justify repressive action and blame the ex-group for social problems (Martín-Baró, 1988). Examples of this can be found in the written press, with statements from authorities labeling the Mapuche social movement as “terrorist” (Álvarez, 2011). Studies in the Colombian context, on the struggles between the state and armed groups, show that the adversary or enemy has been delegitimized and dehumanized through discourses disseminated by the media (Barreto et al., 2009; Gómez et al., 2020). Other studies have found that these actions lead to a lack of support among the general population and may even be held responsible for the damage suffered (Arnoso and Pérez-Sales, 2013). In the Chilean context, the criminalization of the Mapuche people has also been reinforced by the traditional press, which has contributed to or promoted an image of the Mapuche as opponents or enemies of the Chilean government (Rojas and Gálvez, 2018). However, the negative influence of discrimination on distress and well-being was not significant, disconfirming H1 and suggesting that the negative effects of discrimination are weak.

The results of support H2 in relation to experiences of discrimination, collective identity, distress, well-being, and predict participation in social movements. The Anova’s results show that those with a sense of Mapuche ethnicity have a stronger collective identity and are more likely to support for the Mapuche social movement and methods. This evidence is consistent with Smith-Castro (2005), who argue that the perception of discrimination may activate greater group identification which translates into shared beliefs and support for group practices. Collective identity gives a sense of “us,” so when the group is oppressed or ignored, there is an increased commitment and motivation to organize (Javaloy, 2003) and identity processes are a motivation for action to change the situation (Klandermans and Van Stekelenburg, 2020).

Correlation and regression analyses are in line with the previous results and support H2 on psychological well-being, which is positively related to collective identity, although this relationship is weak but with an effect size similar to a meta analysis on ethnic identity and well-being that found an *r* = 0.17.

The relationship between well-being and collective identity would explain group membership as a protective factor where members find social support, cohesion, and a sense of communion together with the possibility of deploying collective coping mechanisms and social participation in the face of a disadvantageous social context (Smith and Silva, 2011; Jetten et al., 2017; Atari and Han, 2018; Bowe et al., 2020).

As for experiences of discrimination, these are positively related to collective identity, and both variables are associated with support for the movement and methods used by the Mapuche movement, as expected. Multiple regression analysis shows that support for the Mapuche movement was specifically predicted by experiences of discrimination and collective identity, confirming H2.

Martín-Baró (1996) proposes that feeling part of a group enhances an individual’s capacity to transform power relations in society, giving rise to a process called “awareness” or the acquisition of knowledge about one’s own identity and the social reality in which one lives. Through active social participation, people seek to deactivate the mechanisms of oppression and dehumanization set in motion by the dominant group (Euzébios Filho, 2010). This explanation would also serve to understand the results of regression analysis, where support for the Mapuche movement presented a positive relationship with two significant collective variables: experiences of discrimination and collective identity.

The moderations reaffirm these same findings and support H3 on the buffering role of collective identity. They show that regardless of experiences of discrimination, low collective identity is associated with greater emotional distress. However, when experiences of discrimination are high, the presence of a stronger collective identity reduces emotional distress, as H3 posits. On the other hand, if experiences of discrimination are low and collective identity is high, then well-being increases. This reaffirms the idea that collective identity acts as a protective factor for mental health (Mossakowski, 2003; Tereucán et al., 2017), mitigating the negative effect of experiences of discrimination on well-being and discomfort. These results are also in line with Haboush-Deloye et al. (2015), who show that strong ethnic identity is associated with lower indicators of suicide and suicidal ideation. Bardol et al. (2020) conclude in a meta-analysis that the sense of affiliation, belonging and social support, that are characteristic of ethnic group membership, are key to mitigating the negative effects of discrimination. Identification with the Mapuche ethnic group provides its members with better resources to cope with the negative effects of acculturative stress and discrimination, as observed in another study by Cheng et al. (2010).

This study presented some important limitations that need to be considered when interpreting these results. First, the sample was not representative, because it was intentionally set up in a stratified way. On the other hand, this study is cross-sectional, which prevents the establishment of cause and effect relationships, analyzing the phenomenon of discrimination in a longitudinal way would be especially interesting since this situation has a long socio-historical background. Thirdly, the ethnic group known as Mestizo includes a heterogeneous group of people, some of them with Mapuche and others Caucasian physical characteristics, which could lead to a difficult interpretation of the results of this group.

In future studies it would be interesting to address variables such as acculturation which refers to the psychological process of adjustment that occurs when different cultures meet (Hjellset and Ihlebæk, 2019). This process implies that people and cultures undergo modifications and accommodation among themselves, and reactions such as rejecting a culture, or implementing adaptation strategies, may occur at an individual or socio-cultural level (Berry, 2005). This variable has been studied especially in the context of migration, where mental health has been linked to the number of perceived social problems, the presence or absence of social support, the type of acculturation strategy implemented, and levels of stress (Yáñez and Cárdenas, 2010). Specifically in Chile, Urzúa et al. (2016) have shown that high levels of stress due to acculturation affect the increase of symptoms associated with mental health problems (Yáñez and Cárdenas, 2010). Tereucán et al. (2017) point out the usefulness of this variable in ethnic contexts, since acculturative stress can arise when people are socially marginalized and are victims of negative attitudes toward their culture of origin, which could also influence suicidal behavior.

This research is consistent with the World Health Organization [WHO] (2008) approach to understanding health through its social determinants such as experiences of poverty and perceived inequality (United Nations Development Programme, 2013). Low levels of participation, cohesion and social integration of people in their environments are key to understanding the persistence of negative indicators in mental health. The findings of this research reinforce the fact that a sense of belonging and attachment to a group can be significant to mental health. The relevance of the identity phenomena of the Mapuche group is related to the broad socio-historical context that leads them to identify as a group in unequal conditions compared to the Caucasian and Mestizo group, as they have suffered constant experiences of violation and discrimination. From this perspective, the need to understand health and well-being from a socio-historical perspective is corroborated (Keyes, 2005; Blanco and Díaz, 2006). Furthermore, it is evident that the processes of redress in vulnerable groups can and should incorporate elements that highlight their identity, seek to eliminate the processes of criminalization that contribute to their discrimination, and guarantee their rights (Virseda et al., 2018). In the case of the Mapuche this is particularly important, as their worldview incorporates the “good life” or in their language “Kume Monguen” as the search for a state of balance between people, their community and their environment. For the Mapuche, people would achieve balance by being in harmony and interpersonal communication in their “lof” (community), their social, productive, cultural, political, environmental, territorial, religious, and cosmic environment (Guajardo, 2017).

This study provides evidence that supports the design of strategies for ensuring mental health through the promotion of collective identity and sense of belonging for the Mapuche people, through activities that encompass their beliefs, traditions, and way of life. The promotion of collective identity constitutes a powerful protective factor for the mental health of individuals and a strategy through which they can confront the systemic inequality, discrimination, and exclusion they experience.

## Data Availability Statement

The raw data supporting the conclusions of this article will be made available by the authors, without undue reservation.

## Ethics Statement

The studies involving human participants were reviewed and approved by the Ethics Committee of the Universidad Santo Tomás. The patients/participants provided their written informed consent to participate in this study.

## Author Contributions

FG proposed the research idea, directed the study, wrote the article, and performed the data analysis and the final review. LV wrote the article, directed the discussion, and participated in the final review. MA, NI, and KS coordinated and performed the data collection in Araucanía region, and contributed to the planning of the study and to the discussion of the results. SG contributed to data analysis and wrote the article and final review.

## Conflict of Interest

The authors declare that the research was conducted in the absence of any commercial or financial relationships that could be construed as a potential conflict of interest.
